# Red-shifted excitation enhances the sensitivity of red genetically encoded Ca^2+^ indicator and enables crosstalk-free two-photon holographic optophysiology

**DOI:** 10.1117/1.JBO.30.11.116003

**Published:** 2025-11-22

**Authors:** Priyanka S. Gore, Masafumi Nishi, Manoj Kumar, Naru Yoneda, Hisao Tsukamoto, Hiroaki Wake, Osamu Matoba, Mitsuhiro Morita

**Affiliations:** a Kobe University, Department of Biology, Kobe, Japan; b Kobe University, Center of Optical Scattering Image Science, Kobe, Japan; c Kobe University, Graduate School of System Informatics, Kobe, Japan; d Nagoya University Graduate School of Medicine, Department of Anatomy and Molecular Cell Biology, Aichi, Japan

**Keywords:** optogenetics, two-photon imaging, genetically-encoded Ca^2+^ indicator, holographic microscopy

## Abstract

**Significance:**

Two-photon (2P) holographic optophysiology, which combines optogenetic actuators and genetically encoded ${\mathrm{Ca}}^{2+}$ indicators (GECIs), enables precise *in vivo* interrogation of neuronal networks. However, this approach is hindered by crosstalk, which is unintentional activation of actuators by imaging light, especially when using blue-light-activated GECIs (e.g., GCaMP) with red-light-activated actuators (e.g., ChRmine).

**Aim:**

To eliminate crosstalk in 2P holographic optophysiology, we employed the inverse combination, namely red GECIs and blue-light-activated actuators and optimized the excitation wavelength, as conventional 2P excitation failed to detect optogenetically induced GECI responses.

**Approach:**

PC12h cells expressing various combinations of GECIs and optogenetic actuators were subjected to simultaneous ${\mathrm{Ca}}^{2+}$ imaging and optogenetic stimulation under both single-photon (1P) and 2P excitation.

**Results:**

Under 1P excitation, crosstalk was evident in the GCaMP6m (blue)/ChRmine (red) pair, but negligible in the R-CaMP1.07 (red)/eTsChR (blue) pair. Under 2P excitation, R-CaMP1.07 showed significantly enhanced sensitivity at a red-shifted wavelength (∼1200  nm) compared with the expected 2P excitation wavelength (1125 nm).

**Conclusion:**

Red-shifted excitation was essential for detecting the small ${\mathrm{Ca}}^{2+}$ elevation following optogenetic stimulation. This optimized condition improves the sensitivity of red GECIs and enables a more robust 2P optophysiology free from crosstalk.

## 1 Introduction

The emerging noninvasive methodology, which combines *in vivo* ${\mathrm{Ca}}^{2+}$ imaging with single-cell-resolution optogenetics,^1^^,^^2^ facilitates the investigation of neural circuits and is referred to as all-optical interrogation of neural circuits^3^^,^^4^ or optophysiology.^5^^–^^7^ This approach is evolving through a collaborative process that integrates innovations from optics and molecular biology. The advent of *in vivo* ${\mathrm{Ca}}^{2+}$ imaging is attributed to the confluence of two-photon (2P) microscopy and genetically encoded ${\mathrm{Ca}}^{2+}$ indicators (GECIs). In addition, single-cell-resolution optogenetics is achieved by the activation of optogenetic actuators derived from microbial opsins using 2P holographic microscopy.^8^^,^^9^ This optical system involves the generation of three-dimensionally distributed holographic actuation sites by 2P stimulation and imaging.^10^^–^^12^ Optophysiology has enabled active investigation of cortical neural activity in behavior^13^ and perception.^14^ It has recently revealed the behavioral consequences of different neuronal activity patterns^15^ and altered functional connectivity in pathological cortical circuits.^16^ Thus, the implementation of optophysiology is anticipated to provide significant advances in basic and clinical neuroscience.

Despite these contributions to neuroscience, technical challenges remain in the practical application of optophysiology using 2P holographic microscopy. The most significant challenge is the unintentional activation of optogenetic actuators by GECI imaging, commonly known as crosstalk.^17^ Among the available GECIs, GFP derivatives, such as GCaMP are the most optimized for protein expression, quantum yield, ${\mathrm{Ca}}^{2+}$-dependent fluorescence changes, and temporal resolution.^18^ Meanwhile, red-shifted derivatives of microbial opsins, such as ChRmine, Crimson, and C1V1 are intensively characterized and optimized as optogenetic actuators for protein expression, current amplitude, and inactivation.^19^ As a result, the combination of GCaMP and red-shifted optogenetic actuators (red actuators) is widely used in optophysiology.^11^^,^^13^^–^^16^ However, this combination carries the risk that the high-energy excitation light used for imaging may inadvertently activate the optogenetic actuators, because GCaMP’s excitation wavelength is shorter than that of red actuators. To mitigate this crosstalk, researchers limit laser power^15^ or set the analysis time window to periods of low actuator expression, either after AAV infection or during the induction of actuator expression. Consequently, these experimental measures employed to avoid the crosstalk compromise the quality of *in vivo* ${\mathrm{Ca}}^{2+}$ imaging and long-term analysis of animal models.

The present study aimed to establish an optophysiology condition with minimal crosstalk. To this end, the crosstalk in the combination of GECI derived from red fluorescent protein (red GECI) and blue-light-activated optogenetic actuator (blue actuator) was compared with that in the combination of GCaMP and red actuator. Because the energy of the excitation light for imaging red GECI is lower than that required for the activation of blue actuator, this combination is likely to be less susceptible to crosstalk. Indeed, optophysiology using red GECIs and blue opsins has been reported in single-photon (1P) systems,^20^^,^^21^ as well as in 2P holographic microscopy systems.^10^^,^^22^ Nevertheless, 6 years after the initial publication of 2P optophysiology using a red GECI and blue opsin combination by Forli et al.^10^ and a subsequent methodology paper employing an improved opsin by the same group,^22^ no other research group has reported either a methodology or a neural network analysis based on this combination to date. We assumed that the papers by Forli et al. did not fully address the methodological requirements for optophysiology, because the majority of the data in these papers consist of electrophysiological characterization of *in vivo* neuronal firing during prolonged 2P holographic stimulation and ${\mathrm{Ca}}^{2+}$ responses were examined under the same stimulation protocol. Therefore, the ${\mathrm{Ca}}^{2+}$ elevations in these papers are nonlinearly associated with action potential generation and reflect the variability in ion channel expression across neurons. To overcome this limitation, the present study optimized the experimental conditions for optophysiology using the undifferentiated PC12h cell line, which is homogeneous and expresses voltage-dependent ${\mathrm{Ca}}^{2+}$ channels but not ${\mathrm{Na}}^{+}$ channels.^23^ This approach is expected to allow quantitative assessment of optophysiology without the nonlinear complexity of neuronal firing and to reveal previously unidentified requirements for optophysiology using red GECI and blue opsin. Furthermore, we conducted a comprehensive and systematic comparison of combinations of a GECI and an actuator and optimized 2P holographic microscopy by employing a tunable laser instead of the widely utilized fixed-wavelength laser. As a result, we found that during two-photon imaging, the sensitivity of red GECIs depends on the excitation wavelength and that small ${\mathrm{Ca}}^{2+}$ elevations induced by optogenetic stimulation are detected only at red-shifted wavelengths relative to conventional excitation. The red-shifted excitation identified in the present study advances 2P holographic microscopy without the crosstalk beyond the previous work by Forli et al. and will accelerate the implementation of optophysiology.

## 2 Materials and Methods

### 2.1 Cell Culture and Physiological Experiments

PC12h cells^24^ were maintained in Dulbecco’s modified Eagle medium (DMEM; 4.5  g/L glucose), supplemented with 5% horse serum (Gibco BRL, Gaithersburg, MD), 5% semi-fetal bovine serum (Mitsubishi Kagaku, Tokyo, Japan), and a penicillin-streptomycin mixture, and incubated in a humidified atmosphere with 5% ${\mathrm{CO}}_{2}$ at 37°C. For imaging experiments, cells were transfected with Lipofectamine 3000 (Thermo Fisher Scientific, Waltham, MA) and seeded onto 12-mm-diameter coverslips precoated with 1 mg/mL polyethyleneimine (Sigma-Aldrich, St Louis, MO). The expression vector for R-CaMP1.07 was constructed by subcloning R-CaMP1.07 (RDB14608, a gift from Dr Junichi Nakai)^20^ into pCX-EGFP, replacing EGFP.^25^ The expression vector for eTsChR, SLF045: AAV-hSyn-eTsChR-GFP,^26^ was a gift from Dr. Adam Cohen (Addgene plasmid # 112280; Ref. 27; RRID:Addgene_112280). The bicistronic expression vector for GCaMP6m and ChRmine, pAAV-hSyn-GCaMP6m-p2A-ChRmine-Kv2.1-WPRE,^14^ and the expression vector for ChR2, pAAV.CAG.hChR2 (H134R)-mCherry.WPRE.SV40, were gifts from Dr. Karl Deisseroth (Addgene plasmid # 131004; Ref. 28; RRID:Addgene_131004 and # 100054; Ref. 29; RRID:Addgene_100054, respectively). The expression vector for jRGECO1a, pAAV.Syn.NES-jRGECO1a.WPRE.SV40,^21^ was a gift from Dr Douglas Kim & GENIE Project (Addgene plasmid # 100854; Ref. 30; RRID:Addgene_100854). The expression vector for human melanopsin with a truncated C-terminus, followed by the 1D4 sequence ETSQVAPA (OPN4dC), was constructed using a mammalian expression vector pMT as described previously.^31^ During imaging, cells were maintained in an extracellular balanced salt solution (BSS) containing 130 mM NaCl, 5.4 mM KCl, 5.5 mM glucose, 2 mM ${\mathrm{CaCl}}_{2}$, 1 mM ${\mathrm{MgCl}}_{2}$, and 20 mM HEPES (pH 7.4). For KCl stimulation, NaCl in the BSS was replaced with KCl to achieve the indicated KCl concentration.

### 2.2 1P Ca^2+^ Imaging and Optogenetic Stimulation

1P imaging was performed using a custom-built microscope equipped with a water-immersion 40× objective lens (LUMPLFLN40xW, Olympus, Tokyo, Japan) and a CMOS camera (C13440-20CU, Hamamatsu Photonics, Hamamatsu, Japan). Data acquisition at 20 Hz was managed using imaging software (HCImage, Hamamatsu). Excitation of GECI or optogenetic actuator activation involved high-power LEDs, SOLIS-470C and SOLIS-565D (Thorlabs Inc, Newton, NJ), connected to a dichroic mirror cube (480/510/570  nm). The duration of optogenetic stimulation was controlled by a pulse generator, PG4000 (Cygnus Tech, Delaware Water Gap, PA), which was connected to the LED driver, DC20 (Thorlabs). The intensities of imaging and stimulation light were measured at the output of the objective lens using a power meter (NOVA II, Ophir, Saitama, Japan) and adjusted as indicated in each figure. The dichroic mirror and emission filter for GCaMP6m imaging were set at 500 and 535/25  nm, whereas those for R-CaMP1.07 imaging were at 575/25 and 620/52  nm.

### 2.3 2P Ca^2+^ Imaging and 2P Holographic Stimulation

**Fig. 1:**
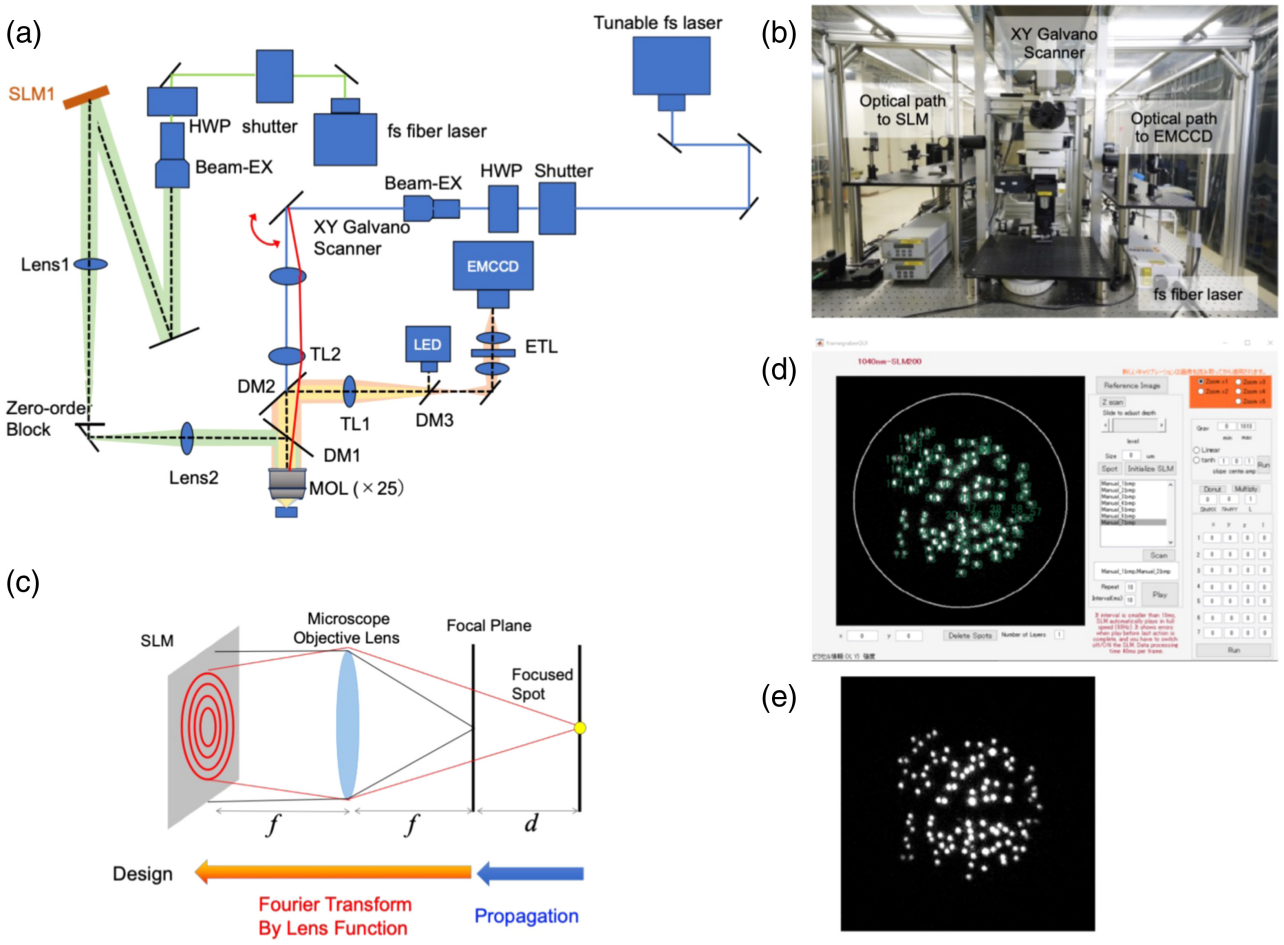
Optical setup of 2P holographic microscope. Schematic diagram of the optical setup integrating holographic stimulation with 2P imaging (a), a photograph of a 2P holographic microscope (b), the calculation process for producing a 3D spot using an SLM (c), the MATLAB-based GUI for generating two-photon spots (d), and a representative fluorescent image (e) are shown.

2P ${\mathrm{Ca}}^{2+}$ imaging and 2P holographic stimulation were performed using a custom-built microscope system, which is shown as a schematic diagram [Fig. 1(a)] and a photograph [Fig. 1(b)]. This holographic microscope system was constructed using a commercially available upright microscope (ECLIPSE FN1, Nikon, Tokyo, Japan) equipped with a water-immersion 25× objective lens (CFI75, Nikon). The system mainly consists of two parts: one is the holographic illumination system using a phase-mode spatial light modulator (SLM), and the other is a three-dimensional fluorescence imaging system using a 2P scanning microscope. In the holographic illumination system, an fematosecond (fs) fiber laser with a central wavelength of 920 nm, a pulse width of 100 fs and a repetition rate of 80 MHz (ALCOR-920, Spark Lasers, Martillac, France) is used. This fs laser light is collimated after the beam expander (Beam-EX) and then illuminates an SLM with a pixel pitch of 8  μm and a resolution of 1920×1080  pixels (SLM-200, Santec, Okayama, Japan). The SLM plane is imaged onto the exit pupil of the microscope objective lens (MOL) by a 4f imaging system using two lenses, Lens1 and Lens2, with focal lengths of 600 mm. The Fourier transformed pattern is generated in the focal plane of the MOL. The illumination beam passes through additional optical components, including dichroic mirrors (DM1, DM2, and DM3), half-wave plates (HWP), tube lenses (TL1 and TL2), an electrically tunable lens (ETL), and a shutter for laser control. Figure 1(c) shows the calculation process for producing a 3D spot using an SLM. When we would like to make N^spots at $\left(u_{k},v_{k},z_{k}\right)$, a complex amplitude distribution at the SLM plane is described as follows.^32^^,^^33^ Horizontal and axial shifts of the focused spot is a linear phase distribution and quadratic phase function, respectively,

$$S(X,Y)=\overset{N}{\underset{k=1}{\sum }}a_{k}\;\exp(-i\frac{2\pi }{\lambda f}(u_{k}X+v_{k}Y))\exp(-i\frac{\pi z_{k}}{\lambda f^{2}}(X^{2}+Y^{2})).$$ (1)

Here, $a_{k}$ denotes the coefficient to be optimized for obtaining the homogeneous spots in the field of view (FOV). In the experiments, only phase distribution is available. Therefore, Eq. (1) is changed to obtain the phase distribution as follows:

$$S_{1}(X,Y)=\exp(i\text{ phase}(X,Y)),$$ (2)

where,

$$\text{phase}(X,Y)=\tan^{-1}(\mathrm{imag}(S(X,Y)),\text{real}(S(X,Y)).$$ (3)

Figure 1(d) shows the MATLAB-based Graphical User Interface (GUI) for creating 2P excitation spots. Here, we briefly introduce the procedure. First, two-dimensional fluorescent images are obtained by a 2P scanning microscope system (C2 plus, Nikon) including control software (NIS Elements, Nikon) and a tunable femtosecond (fs) laser from 660 to 1300 nm at a pulse width of 100 fs and a repetition rate of 80 MHz (Chameleon Discovery NX, Coherent, Saxonburg, PA). Figure 1(e) shows a representative obtained fluorescent image. Then, we select target cells by mouse click to be illuminated by an fs fiber laser at a central wavelength of 920 nm. For 2P GECI imaging with 2P optogenetic stimulation, fluorescence images (128×128  pixels after 8× digital zoom) were acquired at 7 fps, and the duration of holographic illumination was controlled by the shutter placed after the fs fiber laser. For 1P GECI imaging with 2P optogenetic stimulation, fluorescence images (256×256  pixels) were acquired at 9.35 fps using an electron-multiplying charge-coupled device (EMCCD) camera (iXon Ultra, Andor, Concord, MA). The laser spot powers for imaging and stimulation were measured at the output of the objective lens and adjusted as indicated in each figure.

### 2.4 Data Analysis

Images were analyzed using ImageJ to estimate cell activity by selecting regions of interest (ROIs). Statistical analysis was performed using SciPy/Python,^34^ with data expressed as the mean ± standard deviation (SD), and individual cells represented by dots in the plots. Graphs were created using matplotlib/Python.^35^ Differences between groups were assessed using a two-tailed Student’s t-test and ANOVA with Tukey’s post hoc test. Statistical significance was set at P<0.05.

### 2.5 Materials

Nimodipine was obtained from Sigma-Aldrich. All other unspecified chemicals were sourced from Nacalai Tasque (Kyoto, Japan).

## 3 Results

### 3.1 Crosstalk Activation of Optogenetic Actuator by 1P GECI Imaging

**Fig. 2:**
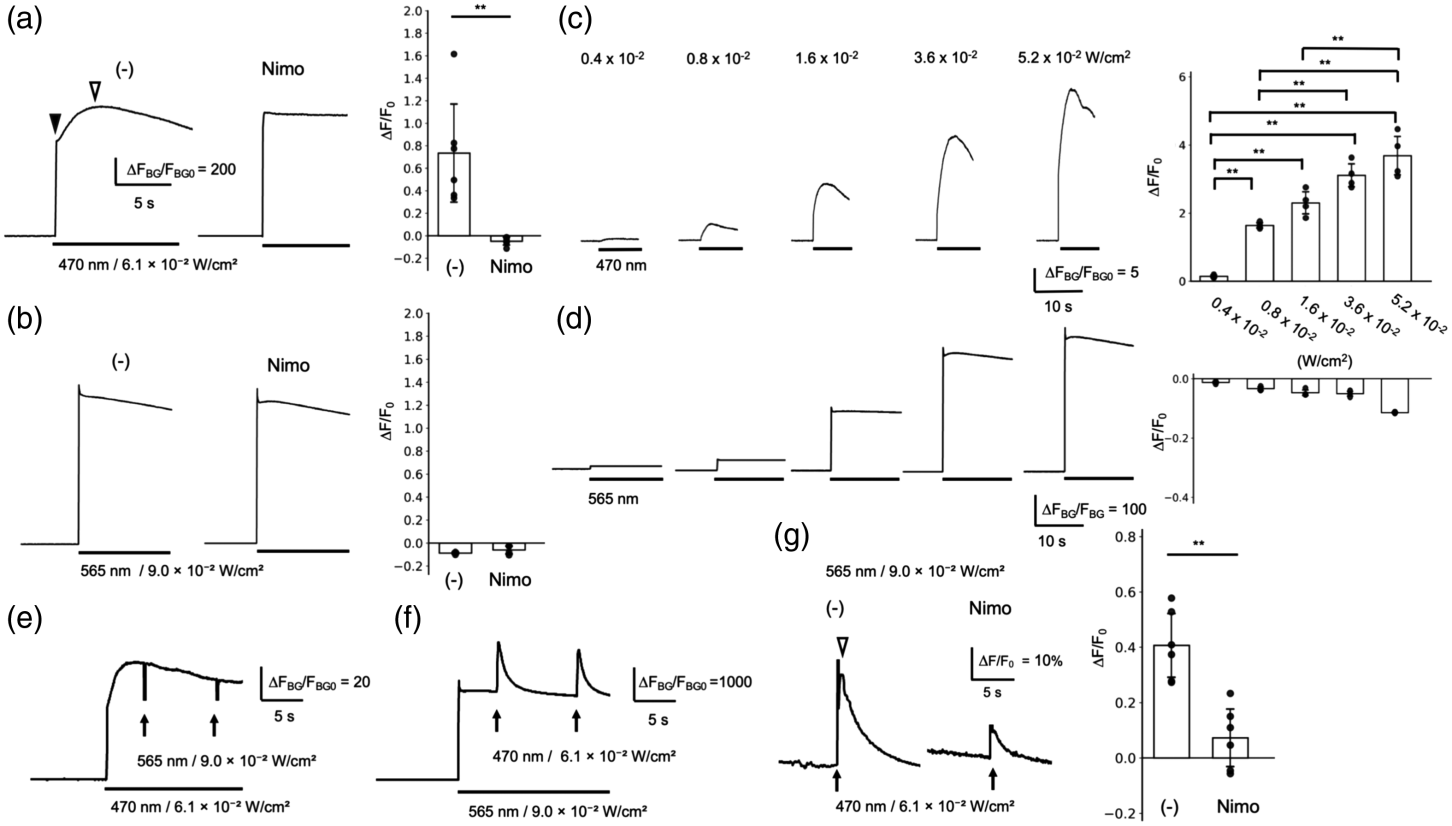
Crosstalk activation of the optogenetic actuator by 1P GECI imaging occurs with GCaMP6m/ChRmine, but not in R-CaMP1.07/eTsChR. PC12h cells expressing GCaMP6m and ChRmine (a), (c), and (e) or R-CaMP1.07 and eTsChR (b), (d), and (f) were subjected to 1P imaging. The wavelengths and powers of excitation light used for GECI imaging and optogenetic stimulation are indicated. The crosstalk activation of the optogenetic actuator was measured as ${\left[{\mathrm{Ca}}^{2+}\right]}_{i}$ elevation due to the activation of L-type voltage-gated ${\mathrm{Ca}}^{2+}$ channels (a), (b). Representative traces of GECI fluorescence are shown on the left and middle. For comparing the fluorescence (F) after excitation (bar), the scale for traces was normalized to the background value before excitation ($F_{BG0}$), according to $\Delta F_{BG}/F_{BG0}=(F-F_{BG0})/F_{BG0}$. Control (–) or 10  μM nimodipine (Nimo). The maximum fluorescence change during excitation due to the crosstalk ($\Delta F/F_{0}$) was calculated from fluorescence intensities at the arrowhead ($F_{0}$) and the empty arrowhead (peak after excitation) and plotted on the right (mean ± *SD*, **p<0.01, t-test). The dependence of crosstalk on excitation intensity for imaging was examined (c), (d). Representative traces of GECI fluorescence before and during excitation at $4.0\times 10^{-3}$ to $5.2\times 10^{-2}\;W/{\mathrm{cm}}^{2}$ (bars), and plots of maximum fluorescence changes during excitation are shown (mean ± *SD*, **p<0.01, ANOVA with Tukey’s post hoc test). The influence of crosstalk on optogenetic responses was examined using 1P stimulation (e), (f). Representative traces of GECI fluorescence in cells subjected to GECI excitation (bars) and optogenetic stimulation by 100 ms 1P illumination (arrows) are shown. The effect of nimodipine on the optogenetic response of R-CaMP1.07/eTsChR was examined (g). Representative fluorescence changes by optogenetic stimulation are shown on the left and middle. The maximum fluorescence change after stimulation artifact is indicated by an empty arrowhead and plotted on the right (mean ± *SD*, **p<0.01, t-test).

The crosstalk activation of optogenetic actuators by 1P GECI imaging was examined in a GCaMP/red actuator combination (GCaMP6m/ChRmine) and a red GECI/blue actuator combination (R-CaMP1.07/eTsChR). The excitation wavelength for GCaMP6m (495 nm)^36^ is shorter than the activation wavelength for ChRmine (585 nm),^14^ whereas the excitation wavelength for R-CaMP1.07 (562 nm)^20^ is longer than the activation wavelength for eTsChR (450 nm).^26^^,^^37^ As illustrated in the activation spectrum shown in Fig. 1(g) of Klapoetke et al.,^37^ all actuators are activated by high-energy short-wavelength light used to excite GCaMP (400 to 500 nm), whereas blue actuators such as ChR2 and TsChR are not activated by low-energy long-wavelength light used to excite red GECIs (>550  nm). Therefore, GCaMP6m/ChRmine, the combination most commonly used in optophysiology,^14^^,^^16^^,^^38^^–^^40^ is likely more susceptible to the crosstalk than R-CaMP1.07/eTsChR. The crosstalk was assessed by fluorescence imaging of PC12h cells expressing optogenetic actuator and GECI, before and during GECI excitation. Because undifferentiated PC12 cells express functional L-type voltage-gated ${\mathrm{Ca}}^{2+}$ channels (VGCCs),^23^ they allow the assessment of depolarization resulting from optogenetic actuator activation by ${\mathrm{Ca}}^{2+}$ imaging using GECI. Following LED excitation at 470 nm, the fluorescence of GCaMP6m/ChRmine gradually increased, and this response was completely inhibited by the L-VGCC blocker nimodipine (10  μM) [Fig. 2(a)]. The peak fluorescence increase, calculated as the ratio ($\Delta F/F_{0}$) between the initial fluorescence value (arrow in [Fig. 2(a)] (–)) and the maximum increase after initiation (open arrow), was 0.74±0.48 in the control group (–), and was completely eliminated, reaching −0.05±0.04 in the nimodipine-treated group (Nimo). These results indicate that the LED illumination used to excite GCaMP6m also activated ChRmine, leading to depolarization and subsequent ${\mathrm{Ca}}^{2+}$ influx through L-type VGCC. By contrast, this crosstalk did not occur with the R-CaMP1.07/eTsChR combination [Fig. 2(b)]. The peak fluorescence increase of R-CaMP1.07/eTsChR was −0.09±0.01 in the control group and −0.06±0.04 in the nimodipine group, indicating that LED initiation did not induce any ${\left[{\mathrm{Ca}}^{2+}\right]}_{i}$ elevation in either group. The crosstalk was further examined using LED illumination ranging from $4.0\times 10^{-3}$ to $5.2\times 10^{-2}\;W/{\mathrm{cm}}^{2}$. The peak fluorescence of GCaMP6m significantly increased from 0.15±0.04 at $4.0\times 10^{-3}\;W/{\mathrm{cm}}^{2}$ to 3.69±0.65 at $5.2\times 10^{-2}\;W/{\mathrm{cm}}^{2}$ [Fig. 2(c)], whereas R-CaMP1.07 fluorescence showed no increase following LED initiation [Fig. 2(d)]. These results indicate that crosstalk was induced under all tested LED conditions in GCaMP6m/ChRmine, but was absent in R-CaMP1.07/eTsChR.

The influence of crosstalk on the optogenetic response was examined by stimulating the optogenetic actuators with 100 ms light pulses. In this study, stimulus light intensity was set at the minimum level required to elicit nearly saturated responses, as this provided relatively stable responses to repeated stimulation. For the GECI–actuator combination that showed no response, representative traces were obtained using higher intensities than those used for the responding combination. The activation of ChRmine at 565 nm caused only brief fluorescence changes, corresponding to stimulation artifacts [Fig. 2(e)]. By contrast, the light pulse at 470 nm elicited a prolonged fluorescence increase in R-CaMP1.07/eTsChR [Fig. 2(f)]. This fluorescence increase was largely attributed to depolarization and subsequent ${\mathrm{Ca}}^{2+}$ influx through L-Type VGCC due to eTsChR activation, because the peak of R-CaMP1.07 fluorescence increase after the artifact (marked by an empty arrowhead) in the control group (0.41±0.13) was significantly suppressed in the nimodipine-treated group (0.07±0.11) [Fig. 2(g)]. These results indicate that GECI imaging induces the crosstalk, eliminating the optogenetic response in GCaMP6m/ChRmine. However, GECI imaging does not affect optogenetic actuator in R-CaMP1.07/eTsChR, thus preserving the optogenetic response.

### 3.2 Crosstalk Activation of Optogenetic Actuator by 2P GECI Imaging

**Fig. 3:**
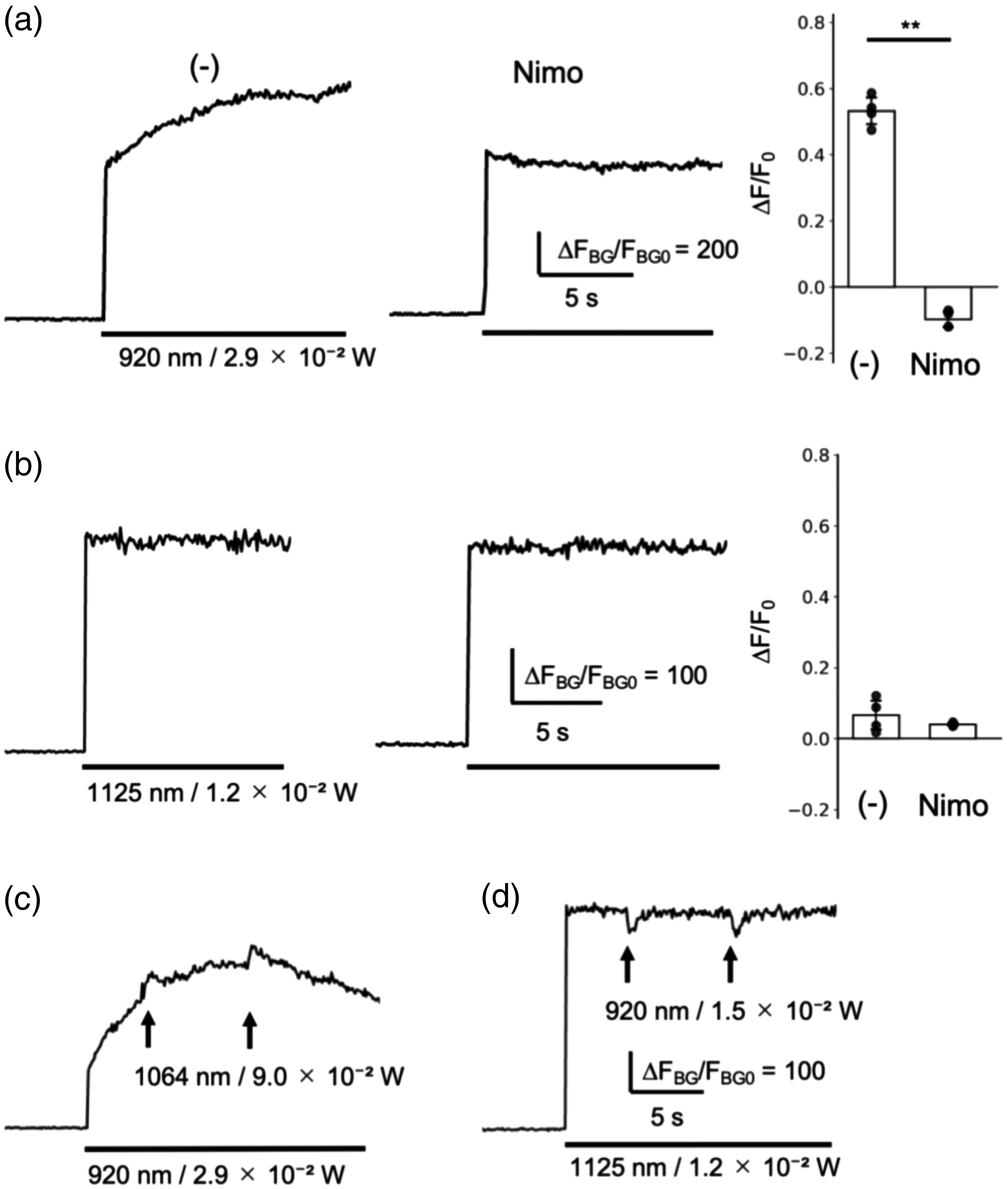
Crosstalk activation of the optogenetic actuator by 2P GECI imaging abolishes optogenetic responses in both GCaMP6m/ChRmine and R-CaMP1.07/eTsChR. PC12h cells expressing GCaMP6m and ChRmine (a), (c) or R-CaMP1.07 and eTsChR (b), (d) were subjected to 2P GECI imaging. The crosstalk activation of the optogenetic actuator was measured as ${\left[{\mathrm{Ca}}^{2+}\right]}_{i}$ elevation (a), (b). Representative traces of GECI fluorescence before and during excitation (bar) in the absence or presence of nimodipine, along with maximum fluorescence changes during excitation (mean ± *SD*, **p<0.01, t-test), are shown. The influence of crosstalk on optogenetic responses was examined using 2P holographic stimulation (c), (d). Representative traces of GECI fluorescence before and during GECI excitation (bars) with 100 ms optogenetic stimulations (arrows) are shown.

**Fig. 4:**
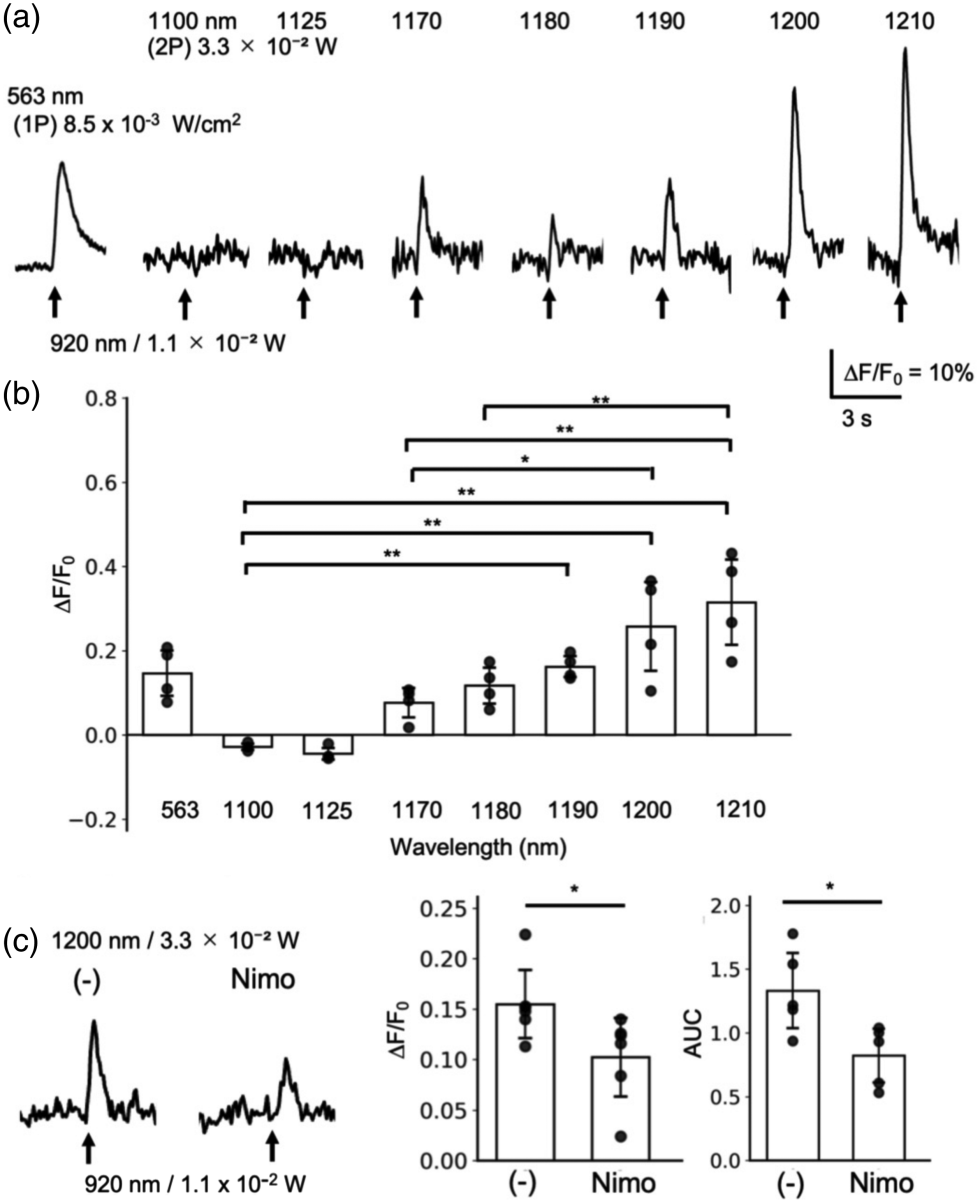
R-CaMP1.07 responses following 2P holographic stimulation to eTsChR depend on the excitation wavelength for imaging. PC12h cells expressing R-CaMP1.07 and eTsChR were imaged with either 1P excitation (563 nm, 1P) or 2P excitation (1100 to 1210 nm) and stimulated with 100 ms 2P holographic spots at 920 nm. Representative traces of R-CaMP1.07 fluorescence and 2P holographic stimulations, marked by arrows, are shown (a). Maximum fluorescence changes were plotted (b) (mean ± SD, *p<0.05, **p<0.01, ANOVA with Tukey’s post hoc test). The effect of nimodipine on the optogenetic response was examined (c). Representative fluorescence changes by holographic stimulation in the absence or presence of nimodipine, along with optogenetic responses quantified as maximum fluorescence change ($\Delta F/F_{0}$) or area under the curve (AUC) (mean ± *SD*, **p<0.01, t-test), are shown.

The crosstalk activation of optogenetic actuator by 2P GECI imaging was examined at the wavelengths of commercially available pulse lasers, 920 nm for GCaMP6m, and at approximately twice the wavelength used for 1P imaging, 1125 nm for R-CaMP1.07. The peak fluorescence increase of GCaMP6m/ChRmine during scanning at 920 nm was 0.53±0.05 in the control group, and it was completely eliminated, reaching −0.10±0.03 in the nimodipine-treated group [Fig. 3(a)]. By contrast, the peak fluorescence increase of R-CaMP1.07/eTsChR during scanning at 1125 nm was 0.07±0.05 in the control group and 0.04±0.01 in the nimodipine group [Fig. 3(b)], indicating that scanning did not elevate $[{\mathrm{Ca}}^{2+}{]}_{i}$. These results indicate that the crosstalk occurs in GCaMP6m/ChRmine, but not in R-CaMP1.07/eTsChR, as with 1P imaging. The influence of crosstalk on the optogenetic response induced by 2P holographic stimulation was examined. 2P stimulation at 1064 nm elicited the fluorescence increase of GCaMP6m/ChRmine, but this response was unlikely to be useful for studying cellular responses due to the unstable baseline fluorescence caused by the crosstalk [Fig. 3(c)]. Unexpectedly, 2P stimulation at 920 nm failed to increase but instead reduced the fluorescence of R-CaMP1.07/eTsChR [Fig. 3(d)]. These negative fluorescence changes, which were not induced by 1P stimulation, are likely attributable to photobleaching during 2P holographic stimulation. Its extent varied between experiments and was occasionally negligible, although the same 2P holographic spots reliably induced ${\mathrm{Ca}}^{2+}$ responses, as shown later (Fig. 4). These results indicate that the optogenetic response to 2P holographic stimulation cannot be detected through 2P GECI imaging using the expected wavelength of twice the excitation wavelength used for 1P ${\mathrm{Ca}}^{2+}$ imaging.

### 3.3 Optimization of the Excitation Wavelength for 2P R-CaMP1.07 Imaging to Obtain the eTsChR Response to 2P Holographic Stimulation

Conditions for 2P imaging were optimized to obtain an optogenetic response following 2P holographic stimulation in R-CaMP1.07/eTsChR. Stimulation at 920 nm induced an R-CaMP1.07 response detectable in 1P imaging with excitation at 563 nm ([Figs. 4(a) and 4(b)] and 563 nm (1P)). Thus, the lack of response in [Fig. 3(d)] is likely due to inadequate imaging conditions rather than to the stimulation protocol. The increase in excitation wavelength for 2P imaging from 1125 nm, approximately double the 563 nm used for 1P imaging, to 1210 nm restored the R-CaMP1.07 response [Figs. 4(a) and 4(b)]. Because the imaging laser power was set to $3.3\times 10^{-2}\;W$ after the objective lens in all tested conditions, this restoration depended solely on the wavelength of the excitation laser for R-CaMP1.07 imaging. The fluorescence did not significantly change at 1100 nm (−0.02±0.01) and 1125 nm (−0.05±0.02). By contrast, it was positive at 1170 nm (0.08±0.04) and significantly increased by 1210 nm (0.32±0.12). In addition, the fluorescence increase at 1200 nm (0.26±0.12) was comparable to that obtained in 1P imaging at 563 nm (0.15±0.06). In the following experiment, 2P R-CaMP1.07 imaging was conducted at 1200 nm because our microscope system had better transmission efficiency at this wavelength than at 1210 nm. The optogenetic response obtained by 2P imaging at 1200 nm was inhibited by nimodipine as in 1P imaging [Fig. 4(c)]. Nimodipine significantly reduced the maximum fluorescence change in the (–) group (0.16±0.04) to that in the Nimo group (0.10±0.04), as well as the area under the curve in the (–) group (1.33±0.33) to that in the Nimo group (0.82±0.23), indicating that the response is mediated by depolarization following optogenetic actuator activation. These results indicate that the eTsChR response to 2P holographic stimulation can be measured through 2P imaging of R-CaMP1.07 when the excitation laser wavelength is set to ∼1200  nm.

### 3.4 Restoration of Optogenetic Response with Optimized Wavelength for 2P Imaging in Other GECI/optogenetic Actuator Combinations

**Fig. 5:**
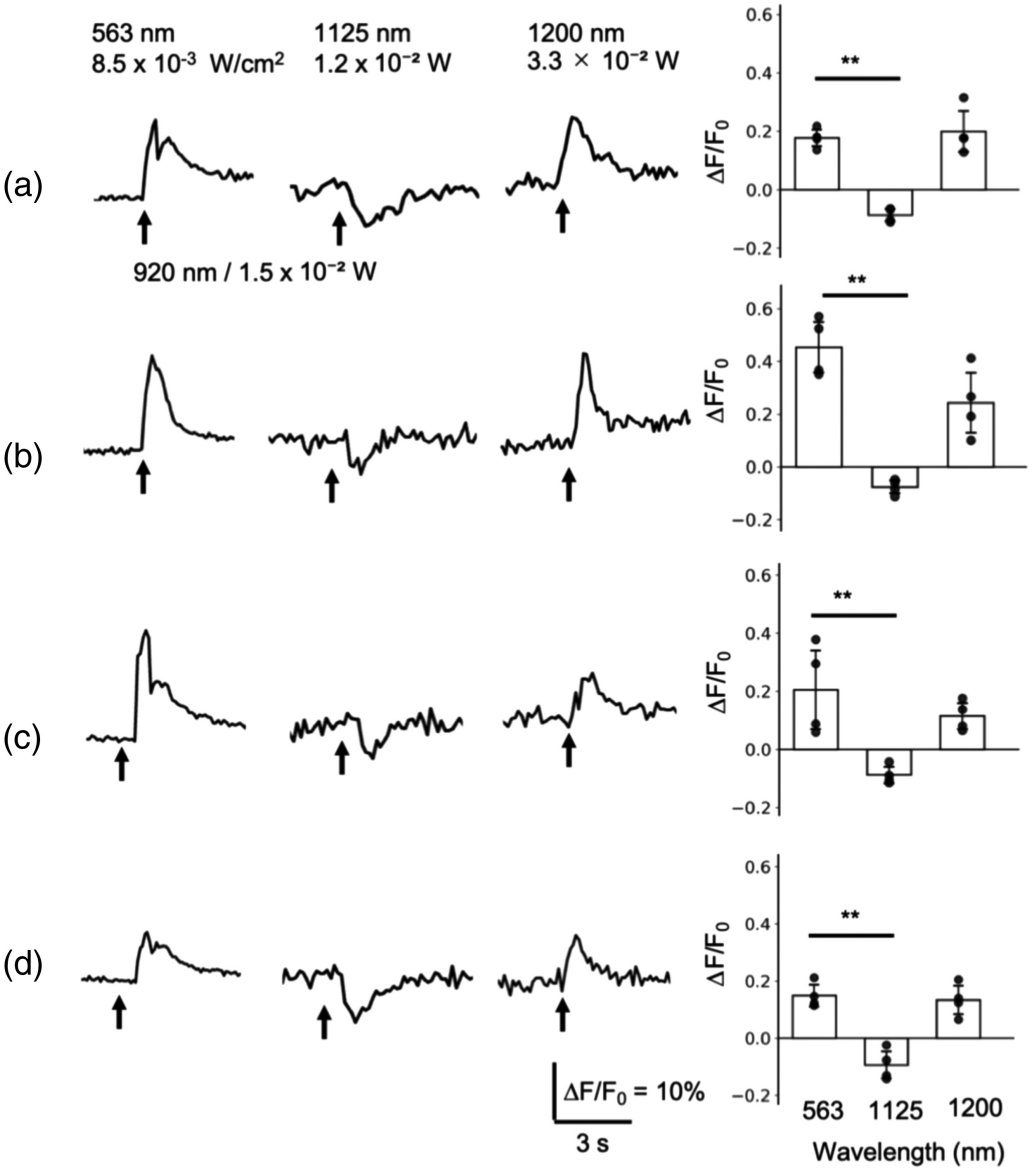
2P imaging at 1200 nm restored the response of red GECIs to 2P holographic stimulation of blue-light absorbing optogenetic actuators. PC12h cells expressing R-CaMP1.07 and eTsChR (a), R-CaMP1.07 and ChR2 (b), R-CaMP1.07 and OPN4dC (c), or jRGECO1a and eTsChR (d) were imaged with either 1P excitation (563 nm) or 2P excitation at 1125 or 1200 nm and stimulated with 100 ms 2P holographic spots at 920 nm. Representative traces of GECI fluorescence excited at the indicated wavelengths and 2P holographic stimulations, marked by arrows, are shown, along with plots of maximum fluorescence changes (mean ± *SD*, *p<0.05, **p<0.01, ANOVA with Tukey’s post hoc test).

The optogenetic response was examined across various GECI/optogenetic actuator combinations using the optimized wavelength for 2P imaging. For this purpose, the most commonly used optogenetic actuator, ChR2,^41^ and a Gq-coupled optogenetic actuator, melanopsin (OPN4dC),^31^ were compared with eTsChR. In addition, another red GECI, jRGECO1a,^21^ was compared with R-CaMP1.07. Consistent with the results in Fig. 4, the eTsChR stimulation at 920 nm induced an equivalent R-CaMP1.07 response in 1P imaging with excitation at 563 nm (0.18±0.03) and in 2P imaging with excitation at 1200 nm (0.20±0.08), whereas a transient reduction in R-CaMP1.07 fluorescence was observed in 2P imaging with excitation at 1125 nm (−0.07±0.04) [Fig. 5(a)]. Similar R-CaMP1.07 responses were elicited by ChR2 stimulation [Fig. 5(b)] and OPN4dC stimulation [Fig. 5(c)]. The peak fluorescence increases at 563 nm (R-CaMP1.07/ChR2, 0.45±0.11; R-CaMP1.07/OPN4dC, 0.21±0.16) were similar to that at 1200 nm (R-CaMP1.07/ChR2, 0.24±0.13; R-CaMP1.07/OPN4dC, 0.12±0.05) but were significantly greater than those at 1125 nm (R-CaMP1.07/ChR2, −0.07±0.03; R-CaMP1.07/OPN4dC, −0.08±0.03). The jRGECO1a response was also restored by using the imaging laser at 1200 nm [Fig. 5(d)]. The peak fluorescence increases at 563 nm (0.15±0.04) were similar to those at 1200 nm (0.13±0.06) while significantly greater than those at 1125 nm (−0.09±0.05). These results indicate that the 2P holographic stimulation is effective for all optogenetic actuators tested. Furthermore, the GECI responses obtained in 1P imaging were restored by increasing the excitation wavelength to 1200 nm in 2P imaging.

### 3.5 KCl-Induced Fluorescence Increases of R-CaMP1.07 Are Not Different Between 1125 and 1200 nm

**Fig. 6:**
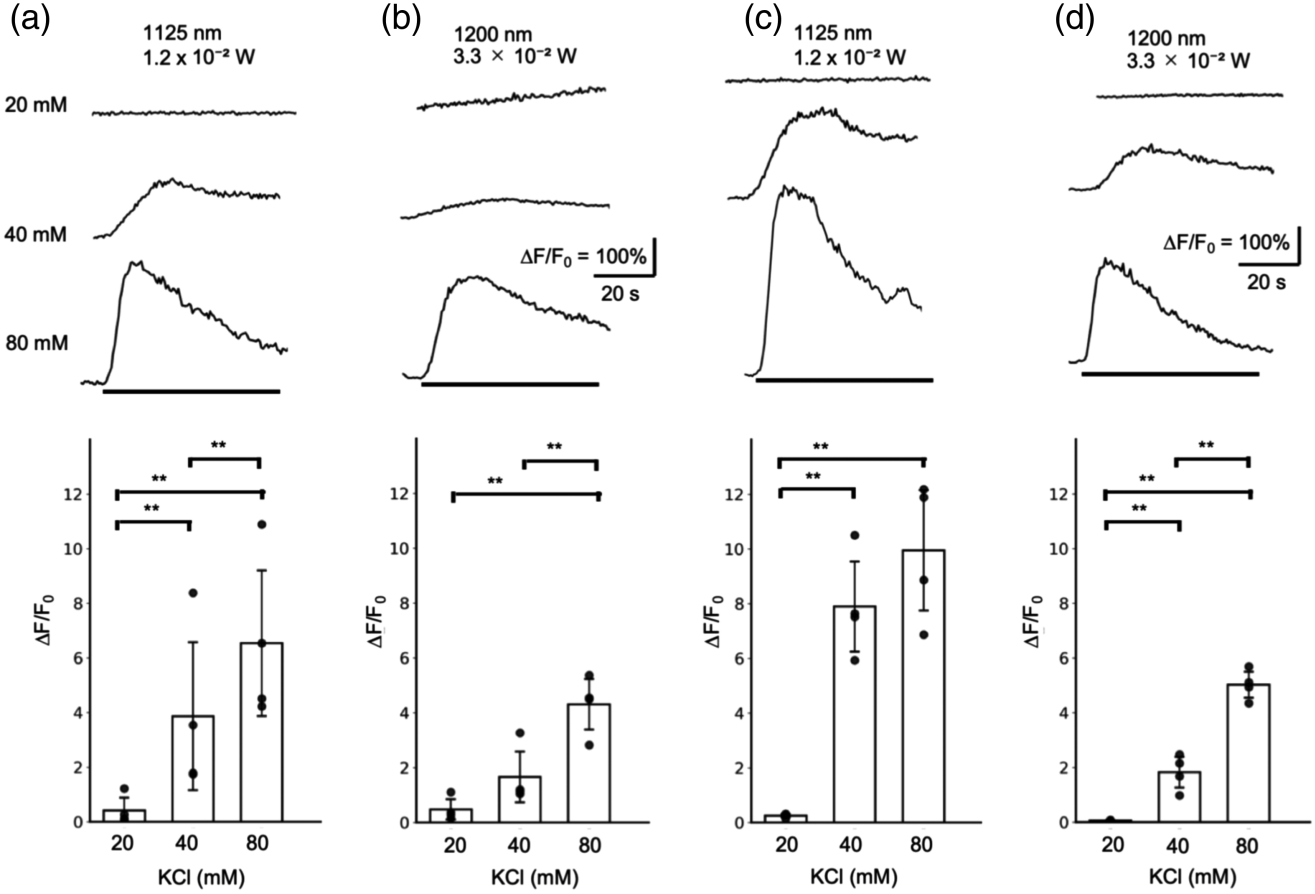
R-CaMP1.07 responses to KCl-induced depolarization were preserved in 2P imaging at both 1125 and 1200 nm. PC12h cells expressing R-CaMP1.07 and eTsChR (a), (b) or R-CaMP1.07 alone (c), (d) were subjected to 2P imaging at either 1125 nm (a), (c) or 1200 nm (b), (d) and stimulated by perfusion with 20, 40, or 80 mM KCl. Representative traces of R-CaMP1.07 fluorescence and KCl stimulations (bars) are shown, along with plots of maximum fluorescence changes (mean ± *SD*, **p<0.01, ANOVA with Tukey’s post hoc test).

To determine whether the lack of optogenetic response at 1125 nm is attributed to the inability of R-CaMP1.07 to detect $[{\mathrm{Ca}}^{2+}{]}_{i}$ elevation or a reduction in sensitivity, the R-CaMP1.07 response to KCl-induced depolarization was compared between 1125 and 1200 nm. PC12h cells expressing R-CaMP1.07/eTsChR or R-CaMP1.07 alone were stimulated with KCl at 20, 40, and 80 mM. The fluorescence changes to 20 mM KCl in R-CaMP1.07/eTsChR at 1125 nm was negligible (0.41±0.55), whereas significantly greater fluorescence increases were induced by 40 mM or 80 mM KCl (3.86±3.12 and 6.54±3.08, respectively) [Fig. 6(a)]. Similar fluorescence changes were induced in R-CaMP1.07/eTsChR at 1200 nm (0.48±0.43 at 20 mM, 1.66±1.07 at 40 mM, and 4.31±1.07 at 80 mM) [Fig. 6(b)]. Furthermore, the responses were consistent in PC12h cells expressing R-CaMP1.07 alone. The responses in R-CaMP1.07 at 1125 nm (0.26±0.08 at 20 mM, 7.90±1.90 at 40 mM, and 9.95±2.54 at 80 mM) [Fig. 6(c)] and those at 1200 nm (0.06±0.01 at 20 mM, 1.82±0.65 at 40 mM, and 5.02±0.55 at 80 mM) [Fig. 6(d)] showed similar fluorescence increases that were dependent on KCl concentration. The fluorescence increases at 40 and 80 mM were approximately tenfold larger than those obtained by optogenetic stimulation in Figs. 4 and 5. Thus, these results indicate that R-CaMP1.07 detects large KCl-induced $[{\mathrm{Ca}}^{2+}{]}_{i}$ elevations at both 1125 and 1200 nm, but small optogenetically induced $[{\mathrm{Ca}}^{2+}{]}_{i}$ elevations only at 1200 nm, suggesting the reduced ${\mathrm{Ca}}^{2+}$ sensitivity of R-CaMP1.07 at 1125 nm. We attempted to establish a pharmacological stimulation method, which reliably induces a small fluorescence increase, similar to optogenetic stimulation, for a detailed analysis of ${\mathrm{Ca}}^{2+}$ sensitivity, but it was not achieved yet.

## 4 Discussion and Conclusion

Because the crosstalk activation of optogenetic actuator by GECI imaging is a major limitation of current optophysiology, the present study aimed to establish an imaging condition to prevent this problem. The commonly used combination of GECI and optogenetic actuator, GCaMP6m/ChRmine, exhibited substantial crosstalk in both 1P and 2P imaging, in line with the experimental constraints of current optophysiology. The prominent fluorescence increases due to the crosstalk, which was observed even at the minimum excitation intensity for 1P imaging [Fig. 2(c)], indicate that it is difficult to avoid crosstalk simply by reducing the excitation intensity. Adjustment of scan parameters (2P excitation volume or dwell time) or optimization of opsin or GECI expression may reduce the crosstalk, but the effect is likely limited. By contrast, no crosstalk was induced in R-CaMP1.07/eTsChR under either imaging condition, and responses to optogenetic stimulation were preserved in 1P imaging. However, no responses to 2P holographic stimulation were obtained in the conventional 2P imaging, where the excitation pulse laser wavelength was set to 1125 nm, approximately twice that of the R-CaMP1.07 excitation wavelength used for 1P imaging. Because the response to 2P optogenetic stimulation was preserved in 1P imaging, it was postulated that the optimization of 2P imaging would restore the response. Indeed, the responses were successfully obtained in the 2P imaging, where the wavelength of the excitation laser was increased to ∼1200  nm. Because the same restoration was found in other combinations of red GECIs and blue-actuators, it was suggested that the wavelength of 2P excitation influences the ${\mathrm{Ca}}^{2+}$ dependency of red GECIs. This influence is likely more prominent in the small $[{\mathrm{Ca}}^{2+}{]}_{i}$ elevations, because KCl-induced $[{\mathrm{Ca}}^{2+}{]}_{i}$ elevations were approximately tenfold larger than optogenetically induced ones, and were detected similarly at both 1125 and 1200 nm. Thus, the sensitivity of red GECIs to ${\mathrm{Ca}}^{2+}$ concentration is likely affected by 2P excitation wavelength. Collectively, the present results suggest that the optophysiology, which uses red GECIs and blue actuators, and in which the excitation wavelength is set to ∼1200  nm, is free from crosstalk and maintains sufficient sensitivity to detect optogenetic responses.

The present study used nimodipine to confirm the depolarization due to optogenetic actuator activation. This blocker completely inhibited the $[{\mathrm{Ca}}^{2+}{]}_{i}$ elevation during GECI imaging in PC12h cells expressing GCaMP6m/ChRmine, but only partially inhibited the $[{\mathrm{Ca}}^{2+}{]}_{i}$ elevation in PC12h cells expressing R-CaMP1.07/eTsChR in response to optogenetic stimulation. This difference is likely due to the inactivation of ${\mathrm{Ca}}^{2+}$ influx pathways. L-type VGCC is the primary VGCC subtype expressed in undifferentiated PC12 cells,^23^ whereas N-type VGCC is additionally expressed^42^ and is upregulated following NGF-induced differentiation.^43^ The ${\mathrm{Ca}}^{2+}$ current through L-type VGCC is sustained with minimal inactivation, whereas that of N-type VGCC is transient with significant inactivation.^44^ In addition to these VGCCs, the $[{\mathrm{Ca}}^{2+}{]}_{i}$ elevation may also be mediated by the modest ${\mathrm{Ca}}^{2+}$ permeability of optogenetic actuators,^45^ whose photocurrents consist of transient and sustained components.^46^ Therefore, it is postulated that the gradual $[{\mathrm{Ca}}^{2+}{]}_{i}$ elevation in GCaMP6m/ChRmine during GECI imaging is primarily attributable to the sustained large ${\mathrm{Ca}}^{2+}$ current through L-type VGCC, which is inhibited by nimodipine. By contrast, the $[{\mathrm{Ca}}^{2+}{]}_{i}$ elevation in response to brief optogenetic stimulation of R-CaMP1.07/eTsChR also involves a transient ${\mathrm{Ca}}^{2+}$ influx mediated by N-type VGCC and/or optogenetic actuator and is therefore only partially inhibited by nimodipine. Although PC12h cells do not express voltage-gated sodium channels and cannot fire action potentials, their expression of VGCCs enables depolarization-induced ${\mathrm{Ca}}^{2+}$ entry that mimics key aspects of neuronal ${\mathrm{Ca}}^{2+}$ signaling. Thus, this model permits controlled evaluation of GECI and actuator performance without the confounding effects of sodium-driven spiking. Because opsins and GECIs behave similarly across cell types due to shared molecular properties, these results are expected to translate to neurons, particularly for detecting subtle ${\mathrm{Ca}}^{2+}$ transients under crosstalk-free conditions.

The restoration of optogenetic response in the 2P imaging of R-CaMP1.07/eTsChR by increasing the excitation wavelength to ∼1200  nm suggests that the excitation wavelength exerts an influence on the ${\mathrm{Ca}}^{2+}$ sensitivity of red GECI. This influence, which was not previously characterized in the *in vitro* spectra under free and saturated ${\mathrm{Ca}}^{2+}$ conditions,^20^^,^^21^ may be attributed to the unknown effects of the intracellular environment on GECI. It is widely acknowledged that there are notable differences in the spectrum and ${\mathrm{Ca}}^{2+}$ dependency of fluorescent ${\mathrm{Ca}}^{2+}$ indicators between *in vitro* and intracellular conditions.^47^ Moreover, it has been proposed that intracellular pH and viscosity may influence the functionality of ${\mathrm{Ca}}^{2+}$ indicators.^48^^,^^49^ In particular, because mApple, from which the red GECI used in this study is derived, exhibits a red-shift in its absorption spectrum with increasing pH,^50^ it is presumed that the red-shift from 1125 to 1200 nm enhances ${\mathrm{Ca}}^{2+}$ detection sensitivity by increasing the fluorescence.

There is no clear literature demonstrating that the red GECI fluorescence generated by excitation at 1200 nm exhibits different properties compared with that generated at 1125 nm. However, a double-humped nature is reported for the 2P excitation spectra of the fluorescence intensity ratios between the ${\mathrm{Ca}}^{2+}$-bound and unbound states of several red GECIs derived from mApple, which is also the source of R-GECO1.07.^51^ This double-humped nature is attributed to the presence of a shoulder in the excitation spectrum under the ${\mathrm{Ca}}^{2+}$-bound condition and to a ${\mathrm{Ca}}^{2+}$-dependent shift in the absorption spectrum. Furthermore, the shift in the absorption spectrum is suggested to be caused by the Stark effect induced by changes in the internal electric field. Notably, most DsRed-derived red fluorescence proteins, including mApple, exhibit double-humped 2P absorption spectra, and only the longer-wavelength peak observed in the 1150 to 1200 nm range is affected by the electric field through the Stark effect.^52^ This is because the longer-wavelength peak corresponds to the purely electronic 0–0 transition, whereas the shorter-wavelength peak corresponds to the 0–1 vibronic transition, which involves vibrational levels and is less directly influenced by the electric field. Therefore, it is assumed that ${\mathrm{Ca}}^{2+}$ binding increases the fluorescence of red GECIs excited at 1125 nm primarily through protein conformational changes, whereas fluorescence excited at 1200 nm changes not only due to conformational changes but also due to the influence of ${\mathrm{Ca}}^{2+}$ on the electric fields around the chromophore. If the electric field positively influences fluorescence, ${\mathrm{Ca}}^{2+}$ would increase fluorescence more efficiently at 1200 nm than at 1125 nm. This may explain why a small ${\mathrm{Ca}}^{2+}$ elevation evoked by optogenetic stimulation was detected only at 1200 nm.

Forli et al.^10^^,^^22^ have published two papers on 2P optophysiology using red GECI and blue actuator. These papers demonstrate that 2P holographic stimulation for 500 ms^10^ or 100 ms^22^ optogenetically induces repetitive action potentials and subsequent $[{\mathrm{Ca}}^{2+}{]}_{i}$ elevation, measured by 2P ${\mathrm{Ca}}^{2+}$ imaging at 1100 nm, in cortical neurons without the crosstalk. However, similar responses were not obtained in our experimental system when the same laser wavelength (1100 nm) was employed for 2P ${\mathrm{Ca}}^{2+}$ imaging. We assume that this discrepancy is explained if the $[{\mathrm{Ca}}^{2+}{]}_{i}$ elevation due to repetitive action potentials in Forli et al. was greater than the $[{\mathrm{Ca}}^{2+}{]}_{i}$ elevation in PC12h cells by our optophysiology system and was measurable even with the detection sensitivity of 2P imaging at 1050 to 1125 nm. This assumption is likely reflected in the current situation that no method or research paper using Forli’s method has been published, even 6 years after the initial publication in 2018.^10^ The $[{\mathrm{Ca}}^{2+}{]}_{i}$ elevation measured by Forli et al. was induced by high-frequency neuronal firing, which may not be achieved in the other research groups. If this is the case, the method proposed in this study will improve the application of optophysiology without the crosstalk. The 2P red GECI imaging at 1200 nm proposed in this study can detect $[{\mathrm{Ca}}^{2+}{]}_{i}$ elevations with higher sensitivity than imaging at 1050 to 1125 nm, particularly at 1100 nm, which was employed by Forli et al.

The present study proposes that an optophysiology condition free from the crosstalk can be achieved using red GECIs and blue actuators, in conjunction with increasing the excitation wavelength for 2P imaging to ∼1200  nm. This approach will advance optophysiology for deeper brain tissue by allowing higher laser power during 2P imaging. This advancement is also facilitated by red GECIs, which have a longer excitation wavelength and are less susceptible to the scattering of the excitation laser within tissue compared with GCaMPs. Nevertheless, the red GECIs have not been optimized to the same degree as GCaMPs, and none of these is capable of detecting action potentials with the same accuracy as GCaMP8.^18^ It is thus anticipated that the present study will facilitate the improvement of red GECIs. In addition to preventing the crosstalk, the present study advances optophysiology by reducing cytotoxicity. It has been reported that repeated light activation of optogenetic actuators can induce cytotoxicity.^53^^,^^54^ Because blue actuators, such as eTsChR and ChR2, generate smaller and faster-decaying photocurrents and are activated by light that is more susceptible to tissue light scattering compared with red actuators, including ChRmine,^55^ they are less effective at depolarizing *in vivo* neurons and thus less cytotoxic. This likely explains why all available transgenic mouse lines, which are generated for optogenetics and both published and listed by Jackson Laboratories, express a blue actuator.^56^^–^^58^ In addition, the cytotoxicity of red actuators likely explains why mice infected with AAV to express these actuators are commonly used during the period of limited actuator expression. Because the methodology proposed in this study uses blue actuators, it can prevent the cytotoxicity issues associated with red actuators. In addition, the compatibility of currently available transgenic mouse lines expressing blue actuators is advantageous for this approach.

We provide a novel solution to the crosstalk, which has hindered the practical application of optophysiology using 2P holographic microscopy. We suppose that the optimal imaging condition found in the present study has been overlooked because commercially available fixed-wavelength pulse lasers cannot perform the optimization described in Fig. 4. Fixed-wavelength fiber lasers and Ti:Sapphire lasers tunable at 920 to 1040 nm are standard for two-photon imaging. In this study, we used a Ti:Sapphire laser with a synchronously pumped optical parametric oscillator to reach longer wavelengths. Although long-wavelength femtosecond lasers remain costly and less available, they are effective for advanced applications and are expected to be more widely adopted. The crosstalk-free, red GECI-based optophysiology demonstrated here may help justify their use, and compact fixed-wavelength lasers at 1200 nm could further improve accessibility. The methodology established in the present study has only been characterized in a model system, PC12h cells, and still needs to be validated *in vivo*. However, because the crosstalk in 2P imaging has been an unsolved problem for a very long time, we believe that even a methodology tested in a model system would make a significant contribution to research in this area. Therefore, this methodology is anticipated to accelerate the implementation of optophysiology using 2P holographic microscopy and advance neuroscience research through this approach.

## Data Availability

Code and data are available from the corresponding author upon reasonable request.

## References

[1] Shemesh O. A. et al., “Temporally precise single-cell-resolution optogenetics,” Nat. Neurosci. 20(12), 1796–1806 (2017). NANEFN 1097-6256 10.1038/s41593-017-0018-8 29184208 PMC5726564

[2] Adesnik H. Abdeladim L., “Probing neural codes with two-photon holographic optogenetics,” Nat. Neurosci. 24(10), 1356–1366 (2021). NANEFN 1097-6256 10.1038/s41593-021-00902-9 34400843 PMC9793863

[3] Emiliani V. et al., “All-optical interrogation of neural circuits,” J. Neurosci. 35(41), 13917–13926 (2015). JNRSDS 0270-6474 10.1523/JNEUROSCI.2916-15.2015 26468193 PMC4604230

[4] Fan L. Z. et al., “All-optical physiology resolves a synaptic basis for behavioral timescale plasticity,” Cell 186(3), 543–559.e19 (2023). CELLB5 0092-8674 10.1016/j.cell.2022.12.035 36669484 PMC10327443

[5] Hagiwara A. et al., “Optophysiological analysis of associational circuits in the olfactory cortex,” Front. Neural Circuits 6, 18 (2012). 10.3389/fncir.2012.00018 22529781 PMC3329886

[r6] Tan P. et al., “Optophysiology: illuminating cell physiology with optogenetics,” Physiol. Rev. 102(3), 1263–1325 (2022). PHREA7 0031-9333 10.1152/physrev.00021.2021 35072525 PMC8993538

[7] Smedemark-Margulies N. Trapani J. G., “Tools, methods, and applications for optophysiology in neuroscience,” Front. Mol. Neurosci. 6, 18 (2013). 10.3389/fnmol.2013.00018 23882179 PMC3713398

[8] Andrasfalvy B. K. et al., “Two-photon single-cell optogenetic control of neuronal activity by sculpted light,” Proc. Natl. Acad. Sci. U. S. A. 107(26), 11981–11986 (2010). 10.1073/pnas.1006620107 20543137 PMC2900666

[9] Papagiakoumou E. et al., “Scanless two-photon excitation of channelrhodopsin-2,” Nat. Methods 7(10), 848–854 (2010).1548-7091 10.1038/nmeth.1505 20852649 PMC7645960

[10] Forli A. et al., “Two-photon bidirectional control and imaging of neuronal excitability with high spatial resolution in vivo,” Cell Rep. 22(11), 3087–3098 (2018). 10.1016/j.celrep.2018.02.063 29539433 PMC5863087

[r11] Yang W. et al., “Simultaneous two-photon imaging and two-photon optogenetics of cortical circuits in three dimensions,” eLife 7, e32671 (2018). 10.7554/eLife.32671 29412138 PMC5832414

[12] Yang W. Yuste R., “Holographic imaging and photostimulation of neural activity,” Curr. Opin. Neurobiol. 50, 211–221 (2018). COPUEN 0959-4388 10.1016/j.conb.2018.03.006 29660600

[13] Carrillo-Reid L. et al., “Controlling visually guided behavior by holographic recalling of cortical ensembles,” Cell 178(2), 447–457.e5 (2019). CELLB5 0092-8674 10.1016/j.cell.2019.05.045 31257030 PMC6747687

[r14] Marshel J. H. et al., “Cortical layer-specific critical dynamics triggering perception,” Science 365(6453), eaaw5202 (2019). SCIEAS 0036-8075 10.1126/science.aaw5202 31320556 PMC6711485

[r15] Rowland J. M. et al., “Propagation of activity through the cortical hierarchy and perception are determined by neural variability,” Nat. Neurosci. 26(9), 1584–1594 (2023). NANEFN 1097-6256 10.1038/s41593-023-01413-5 37640911 PMC10471496

[16] Okada T. et al., “Pain induces stable, active microcircuits in the somatosensory cortex that provide a therapeutic target,” Sci. Adv. 7(12), eabd8261 (2021). STAMCV 1468-6996 10.1126/sciadv.abd8261 33741588 PMC7978434

[17] Rindner D. J. Lur G., “Practical considerations in an era of multicolor optogenetics,” Front. Cell. Neurosci. 17, 1160245 (2023). 10.3389/fncel.2023.1160245 37293628 PMC10244638

[18] Zhang Y. et al., “Fast and sensitive GCaMP calcium indicators for imaging neural populations,” Nature 615(7954), 884–891 (2023). 10.1038/s41586-023-05828-9 36922596 PMC10060165

[19] Lehtinen K. Nokia M. S. Takala H., “Red light optogenetics in neuroscience,” Front. Cell. Neurosci. 15, 778900 (2022). 10.3389/fncel.2021.778900 35046775 PMC8761848

[20] Ohkura M. et al., “An improved genetically encoded red fluorescent ${\mathrm{Ca}}^{2+}$ indicator for detecting optically evoked action potentials,” PLoS One 7(7), e39933 (2012). POLNCL 1932-6203 10.1371/journal.pone.0039933 22808076 PMC3393713

[21] Dana H. et al., “Sensitive red protein calcium indicators for imaging neural activity,” eLife 5, e12727 (2016). 10.7554/eLife.12727 27011354 PMC4846379

[22] Forli A. et al., “Optogenetic strategies for high-efficiency all-optical interrogation using blue-light-sensitive opsins,” eLife 10, e63359 (2021). 10.7554/eLife.63359 34032211 PMC8177884

[23] Avidor B. et al., “Cardiac L-type ${\mathrm{Ca}}^{2+}$ channel triggers transmitter release in PC12 cells,” FEBS Lett. 342(2), 209–213 (1994). FEBLAL 0014-5793 10.1016/0014-5793(94)80502-4 8143879

[24] Hatanaka H., “Nerve growth factor-mediated differentiation of a nerve cell line cultured in a hormone-supplemented serum-free medium,” Dev. Brain Res. 6(3), 243–250 (1983). DBRRDB 0165-3806 10.1016/0165-3806(83)90063-9 6831247

[25] Niwa H. Yamamura K. Miyazaki J., “Efficient selection for high-expression transfectants with a novel eukaryotic vector,” Gene 108(2), 193–199 (1991). GENED6 03781119 10.1016/0378-1119(91)90434-D 1660837

[26] Farhi S. L. et al., “Wide-area all-optical neurophysiology in acute brain slices,” J. Neurosci. 39(25), 4889–4908 (2019). JNRSDS 0270-6474 10.1523/JNEUROSCI.0168-19.2019 30952812 PMC6670252

[27] http://n2t.net/addgene:112280.

[28] http://n2t.net/addgene:131004.

[29] http://n2t.net/addgene:100054.

[30] http://n2t.net/addgene:100854.

[31] Tsukamoto H. et al., “Retinal attachment instability is diversified among mammalian melanopsins,” J. Biol. Chem. 290(45), 27176–27187 (2015). JBCHA3 0021-9258 10.1074/jbc.M115.666305 26416885 PMC4646394

[32] Daria V. et al., “Arbitrary multisite two-photon excitation in four dimensions,” Appl. Phys. Lett. 95, 093701 (2009). APPLAB 0003-6951 10.1063/1.3216581

[33] Go M. A. et al., “Simultaneous multi-site two-photon photostimulation in three dimensions,” J. Biophotonics 5(10), 745–753 (2012). 10.1002/jbio.201100101 22345073

[34] Virtanen P. et al., “SciPy 1.0: fundamental algorithms for scientific computing in Python,” Nat. Methods 17(3), 261–272 (2020).1548-7091 10.1038/s41592-019-0686-2 32015543 PMC7056644

[35] Hunter J. D., “Matplotlib: a 2D graphics environment,” Comput. Sci. Eng. 9(3), 90–95 (2007). 10.1109/MCSE.2007.55

[36] Chen T. W. et al., “Ultrasensitive fluorescent proteins for imaging neuronal activity,” Nature 499(7458), 295–300 (2013). 10.1038/nature12354 23868258 PMC3777791

[37] Klapoetke N. C. et al., “Independent optical excitation of distinct neural populations,” Nat. Methods 11(3), 338–346 (2014).1548-7091 10.1038/nmeth.2836 24509633 PMC3943671

[38] Russell L. E. et al., “All-optical interrogation of neural circuits in behaving mice,” Nat. Protoc. 17(7), 1579–1620 (2022).1754-2189 10.1038/s41596-022-00691-w 35478249 PMC7616378

[r39] Sylwestrak E. L. et al., “Cell-type-specific population dynamics of diverse reward computations,” Cell 185(19), 3568–3587.e27 (2022). CELLB5 0092-8674 10.1016/j.cell.2022.08.019 36113428 PMC10387374

[40] Yokoyama T. et al., “A multicolor suite for deciphering population coding of calcium and cAMP in vivo,” Nat. Methods 21(5), 897–907 (2024).1548-7091 10.1038/s41592-024-02222-9 38514778 PMC11093745

[41] Adamantidis A. et al., “Optogenetics: 10 years after ChR2 in neurons—views from the community,” Nat. Neurosci. 18(9), 1202–1212 (2015). NANEFN 1097-6256 10.1038/nn.4106 26308981

[42] Janigro D. Maccaferri G. Meldolesi J., “Calcium channels in undifferentiated PC12 rat pheochromocytoma cells,” FEBS Lett. 255(2), 398–400 (1989). FEBLAL 0014-5793 10.1016/0014-5793(89)81131-7 2551740

[43] Grantham C. J. Main M. J. Cannell M. B., “Fluspirilene block of N-type calcium current in NGF-differentiated PC12 cells,” Br. J. Pharmacol. 111(2), 483 (1994). 10.1111/j.1476-5381.1994.tb14762.x 8004393 PMC1909950

[44] Stotz S. C. Zamponi G. W., “Identification of inactivation determinants in the domain IIS6 region of high voltage-activated calcium channels,” J. Biol. Chem. 276(35), 33001–33010 (2001). JBCHA3 0021-9258 10.1074/jbc.M104387200 11402052

[45] Fernandez Lahore R. G. et al., “Calcium-permeable channelrhodopsins for the photocontrol of calcium signalling,” Nat. Commun. 13(1), 7844 (2022). NCAOBW 2041-1723 10.1038/s41467-022-35373-4 36543773 PMC9772239

[46] Bansal H. Pyari G. Roy S., “Theoretical prediction of broadband ambient light optogenetic vision restoration with ChRmine and its mutants,” Sci. Rep. 14(1), 11642 (2024). SRCEC3 2045-2322 10.1038/s41598-024-62558-2 38773346 PMC11109128

[47] Moore E. D. et al., “${\mathrm{Ca}}^{2+}$ imaging in single living cells: theoretical and practical issues,” Cell Calcium 11(2–3), 157–179 (1990). CECADV 0143-4160 10.1016/0143-4160(90)90068-6 2191780

[48] Grynkiewicz G. Poenie M. Tsien R. Y., “A new generation of ${\mathrm{Ca}}^{2+}$ indicators with greatly improved fluorescence properties,” J. Biol. Chem. 260(6), 3440–3450 (1985). JBCHA3 0021-9258 10.1016/S0021-9258(19)83641-4 3838314

[49] Poenie M., “Alteration of intracellular Fura-2 fluorescence by viscosity: a simple correction,” Cell Calcium 11(2–3), 85–91 (1990). CECADV 0143-4160 10.1016/0143-4160(90)90062-Y 2354506

[50] Sartor A. M. et al., “Characterization of mApple as a red fluorescent protein for cryogenic single-molecule imaging with turn-off and turn-on active control mechanisms,” J. Phys. Chem. B 127(12), 2690–2700 (2023). JPCBFK 1520-6106 10.1021/acs.jpcb.2c08995 36943356 PMC10069424

[51] Molina R. S. et al., “Understanding the fluorescence change in red genetically encoded calcium ion indicators,” Biophys. J. 116(10), 1873–1886 (2019). BIOJAU 0006-3495 10.1016/j.bpj.2019.04.007 31054773 PMC6531872

[52] Drobizhev M. et al., “Color hues in red fluorescent proteins are due to internal quadratic Stark effect,” J. Phys. Chem. B 113(39), 12860–12864 (2009). JPCBFK 1520-6106 10.1021/jp907085p 19775174 PMC2893592

[53] Li Q. et al., “Electrophysiological properties and viability of neonatal rat ventricular myocyte cultures with inducible ChR2 expression,” Sci. Rep. 7, 1531 (2017). SRCEC3 2045-2322 10.1038/s41598-017-01723-2 28484220 PMC5431527

[54] Perny M. et al., “Chronic activation of the D156A point mutant of channelrhodopsin-2 signals apoptotic cell death: the good and the bad,” Cell Death Dis. 7(11), e2447 (2016). 10.1038/cddis.2016.351 27809305 PMC5260891

[55] Sridharan S. et al., “High-performance microbial opsins for spatially and temporally precise perturbations of large neuronal networks,” Neuron 110(7), 1139–1155.e6 (2022). NERNET 0896-6273 10.1016/j.neuron.2022.01.008 35120626 PMC8989680

[56] Madisen L. et al., “A toolbox of Cre-dependent optogenetic transgenic mice for light-induced activation and silencing,” Nat. Neurosci. 15(5), 793–802 (2012). NANEFN 1097-6256 10.1038/nn.3078 22446880 PMC3337962

[r57] Bounds H. A. et al., “All-optical recreation of naturalistic neural activity with a multifunctional transgenic reporter mouse,” Cell Rep. 42(8), 112909 (2023). 10.1016/j.celrep.2023.112909 37542722 PMC10755854

[58] Iwai Y. et al., “Transient astrocytic Gq signaling underlies remote memory enhancement,” Front. Neural Circuits 15, 658343 (2021). 10.3389/fncir.2021.658343 33828463 PMC8019746

